# Absolute contrast estimation for soft X-ray photon fluctuation spectroscopy using a variational droplet model

**DOI:** 10.1038/s41598-021-98774-3

**Published:** 2021-09-30

**Authors:** N. G. Burdet, V. Esposito, M. H. Seaberg, C. H. Yoon, J. J. Turner

**Affiliations:** 1 Stanford Institute for Materials and Energy Sciences, Stanford University and SLAC National Accelerator Laboratory, Menlo Park, CA 94025 USA; 2 Linac Coherent Light Source, SLAC National Accelerator Laboratory, Menlo Park, CA 94025 USA

**Keywords:** Scientific data, Characterization and analytical techniques

## Abstract

X-ray photon fluctuation spectroscopy using a two-pulse mode at the Linac Coherent Light Source has great potential for the study of quantum fluctuations in materials as it allows for exploration of low-energy physics. However, the complexity of the data analysis and interpretation still prevent recovering real-time results during an experiment, and can even complicate post-analysis processes. This is particularly true for high-spatial resolution applications using CCDs with small pixels, which can decrease the photon mapping accuracy resulting from the large electron cloud generation at the detector. Droplet algorithms endeavor to restore accurate photon maps, but the results can be altered by their hyper-parameters. We present numerical modeling tools through extensive simulations that mimic previous x-ray photon fluctuation spectroscopy experiments. By modification of a fast droplet algorithm, our results demonstrate how to optimize the precise parameters that lift the intrinsic counting degeneracy impeding accuracy in extracting the speckle contrast. These results allow for an absolute determination of the summed contrast from multi-pulse x-ray speckle diffraction, the modus operandi by which the correlation time for spontaneous fluctuations can be measured.

## Introduction

Spontaneous nanoscale fluctuations manifest over a wide range of material systems and X-FELs offer a new range of time and energy scales over which the effect of these fluctuations on material properties can be studied. In x-ray photon correlation spectroscopy (XPCS), the sample dynamics are revealed by correlating a time sequence of images emanating from the coherent scattering of a pulse train^1,2^. More specifically, the minute changes with time delay $\tau$, are captured by the function $g^{(2)}(q, \tau )$, which measures the temporal auto-correlation of the scattered intensity $I(q, \tau )$. The sensitivity comes from the coherent nature of the scattered photons, which interfere to forge a speckle pattern that uniquely corresponds to the fingerprint of the sample complex domain distribution.

New versions of XPCS using a split-pulse or a double-bunch pulse pair, what we refer to here as x-ray photon fluctuation spectroscopy (XPFS), are methodologies which are being developed in which the photons are not correlated, but counted. The coherent photon distribution follows a well-subscribed recipe which can be related back to what is known as the contrast function, and hence $g^{(2)}(q, \tau )$. In other words, the photon fluctuations finally yield the photon correlation function. This has been recently demonstrated^3^ to remove the detector read-out limitations that limit the shortest measurement times in XPCS. It relies on measuring the change in contrast $C(q, \tau )$ from the recorded sum of two diffracted patterns delayed by a variable time $\tau$, which has been proven equivalent to measuring $g^{(2)}(q, \tau )$, the intensity-intensity correlation function^4^.

The two x-ray pulses can be created in a variety of ways. For example, they can be obtained from splitting a single coherent pulse by means of an optical setup^5^, splitting the electron pulse by slicing before the x-rays are generated^6^, or generating the two at the source with two separate laser systems to create two electron pulses using the two-bucket mode^7^. These different methods cover the range from tens of fs to or order one microsecond, bridging the gap with the timescales available at high-brightness synchrotron sources. XPFS opens up a new realm of energy ranges corresponding to collective excitations in condensed matter.

While it is the unique combination of coherence and the ultra-short pulse duration of the photons produced by X-FELs that foster this technique, with a time resolution orders of magnitude faster than that available with current detector read-out capabilities, the high fluence that results can potentially induce out-of-equilibrium dynamics in the system being probed. In order to preserve the system from this effect, the beam intensity has to be drastically reduced to the weak photon counting limit^3^. In this sparse photon regime, the intrinsic sample dynamics are deduced by invoking a photon statistical tool to analyze single shot detector images.

The fitting statistics to reliably extract $C(q, \tau )$ is established through hundreds or thousands of frames using the formalism of quantum optics. Mathematically, this is carried out via a negative binomial distribution^8^ which contains the contribution from the expected scattering (Gamma distribution) and the shot-noise (Poisson distribution), convolved into a single function:

1 $$\begin{aligned} P(k, \overline{k}, M) = \frac{\Gamma (k+M)}{k! \Gamma (M)} \left( \frac{\overline{k}}{\overline{k}+M}\right) ^k\left( \frac{M}{\overline{k}+M}\right) ^M , \end{aligned}$$

where $\Gamma (x)$ is the gamma function, *k* is an integer number of photons per speckle, $\overline{k}$ the average number of photons per speckle, and *M* is the number of modes (degrees of freedom) in the speckle pattern, inversely related to the contrast by $C= 1 / \sqrt{M}$. Note that the traditional definition for contrast in XPCS is sometimes referred to as $\beta = 1 / M$, where $\beta=C^2$ in our case. For a given contrast and mean intensity, this function gives the probability of counting *k* photons per speckle. Experimentally, the starting zero delay point, $C_{0}(q,\tau = 0)$ is expected to be near unity, but can differ ($M > 1$) depending on the transverse and longitudinal coherence.

This is especially true for large scattering angles, where the longitudinal coherence is reduced, and can contain an increased number of modes. For $\tau$ > 0, the contrast is a measure of the sample dynamics. The delay time $\tau$ is increased until it is larger than the correlation time, at which point the two speckle pattern reduce to a contrast of $C_{0}/\sqrt{2}$, for full decorrelation^8^. The speckle size is given by $s=\lambda d/l$ where $\lambda$ is the wavelength, *l* is the distance to detector and *d* the beam size. Since $\lambda$ is usually dictated by the sample of interest in the soft x-ray range for resonant scattering, given a finite pixel size, oversampling of the speckles is achieved by varying the beam size or the distance from the sample to the detector, since the beam is fully coherent.

One of the critical issues when using pixelated detectors, is the electron cloud generated around each detected photon hit due to the charge sharing. Depending on the x-ray energy and the size of pixels, the clouds can overlap and eventually coalesce to form islands. Furthermore, readout noise can extend the blurring in a non-trivial way as well. Since a wrong registration of the photon for a given speckle will have a strong impact on the determination of the contrast, a “deblurring” method called a droplet algorithm has been applied to decipher the effect of charge sharing on every image. Though other authors have studied optimization of droplet algorithms^9^, the prescriptions mainly apply to the situation where charge clouds are small compared to the pixels. The opposite situation however was met in the experiment by Seaberg et al.^3^, the first proof-of-principle of XPFS using the double pulse summed-contrast method carried out at the LCLS^10^. In that case, an Andor CCD camera bearing a small pixel size of 13 $\mu$m was employed to achieve sufficient oversampling. This leaves a strenuous task for the droplet algorithm in the disentangling of large charge clouds that overflow into neighboring pixels, and with it the many algorithm parameters that can compromise a full restoration of the photon map.

In this paper, we demonstrate a solution to this problem for incorporating a variational droplet algorithm for high resolution pixelated detectors used in XPFS. By modeling this experiment with a detailed account of the detector characteristics, extensive simulations have been carried out in order to determine the precise algorithmic parameters that extract the true absolute contrast. The limit of the average photon intensity in which the algorithm operates best is also examined. Finally, additional photon energies are analyzed in view of future experiments to study fluctuations at XFELs.

## Model

Experimental data are modeled by simulating pixelated grids with weak intensity speckle patterns using photon statistics following a negative binomial distribution (Eq. 1), and onto which Gaussian shaped charge clouds are convolved with each photon, to account for charge sharing effects on the CCD detector:

2 $$\begin{aligned} g(x,y) = \frac{1}{2 \pi \sigma _{G}^2} \exp \left( -\frac{ (x-x_c)^2 }{2 \sigma _{G}^2} - \frac{ (y-y_c)^2 }{2 \sigma _{G}^2} \right) , \end{aligned}$$

where $\sigma _{G}$ is the radius of the charge cloud, also referred to as the strength of the charge sharing parameter. The variables *x* and *y* are the pixel coordinate and ($x_c$, $y_c$) is the random location center of the charge cloud within that pixel. In addition, pixels receive a dose of readout noise along with a small gain non-uniformity.

A similar model was already successfully applied in the work of Lehmkuhler et al.^11^ with which they were able to reproduce an experimental histogram from data taken at the SACLA free-electron laser and thereby deduce the spatial coherence, and the resultant contrast, by simply adjusting the model parameters and fitting. However, for larger charge cloud probability (or smaller pixels) as found in the experiment discussed here, a simple contrast interpretation from the histogram can become ambiguous and calls for a more rigorous analysis of the charge cloud maps using a droplet algorithm.

**Figure 1:**
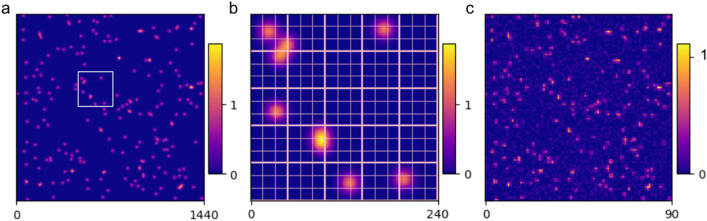
CCD charge sharing model of speckle patterns. (**a**) Sparse distribution of weakly scattered photons on a $3\times 3$ pixels/speckle basis, generated via the negative binomial distribution (Eq. 1) within the range $\overline{k}= [0.025, 0.25]$ photons/speckle. The selected contrast is 0.7. The map is high-resolution since every pixel is sub-divided by $16\times 16$ to obtain a sub-pixel placement of Gaussian shaped charge clouds (FWHM $\sim 1$ pixel). (**b**) Enlarged portion of the high resolution photon map (white square box in **a**), displaying a $5\times 5$ speckle area (bold lines boundaries), each speckle containing $3\times 3$ pixels (light lines boundaries). Photons can sprawl over both pixel and speckle boundaries. (**c**) Final ccd image of the speckle pattern obtained after binning the high resolution photon map (**a**) to a coarser $90\times 90$ grid and adding readout noise ($\sigma _N = 0.05$ photon). A single photon readout is 350 ADU.

To this aim, photon maps of known contrast are generated from a random sampling of the negative binomial distribution (Eq. 1) in the weak scattered photon limit. Speckle patterns are first simulated with an average photon count rate on the interval $\overline{k} = [0.025, 0.25]$ photon/speckle, and expanded on a $90 \times 90$ pixel basis and with a nominal speckle size of $\sim$ $3\times 3$ pixels. This particular $\overline{k}$ range accounts for the inexorable stochastic shot-to-shot intensity fluctuation of the XFEL beam, while also being within the sparse photon limit. The beam fluctuation distribution would likely follow a Gamma distribution without undergoing scattering from the sample. Each pixel is then sub-divided into a $16\times 16$ array to attain sub-pixel resolution in the placement of the random photon location within each speckle. Once a photon is allocated to a speckle, it is randomly placed within the corresponding $3\times 3$ pixel/speckle area, according to a homogeneous distribution within each speckle (Fig. 1a). Note the precise distribution within a speckle is not important, as only the number of photons per speckle affects the physical measurement. By convolving these high-resolution maps with Gaussian distributed charge clouds (Eq. 2) of size $\sigma _{G}=0.45 \pm {0.0125}$ pixels, strong charge sharing is enabled (Fig. 1b). The intensity of a single charge cloud is set to an ADU of 350. Both values were taken from the experimental data in Seaberg et al.^3^, which used a photon energy of 1.19 keV. Finally, to emulate the experimental data, the high-resolution map is binned back by $16\times 16$ and Gaussian noise of width $\sigma _{N}=0.05$ photon, centered around zero, is added to every pixel along with a non-uniformity gain parameter $\sigma _r$=0.02 (Fig. 1c). In this section, we will analyze whether this prototypical model 1 is sufficient to reproduce the experimental data.

**Figure 2:**
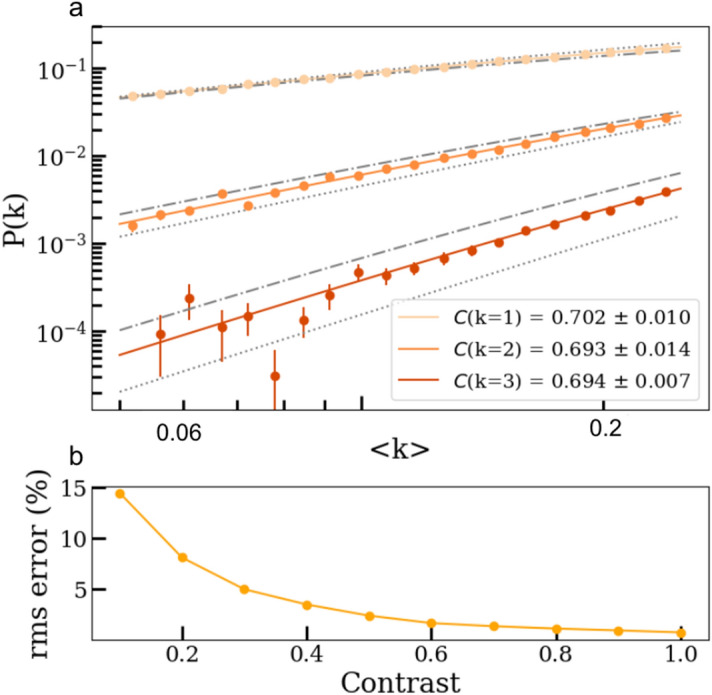
Fitting of observed probabilities P(k) for k = 1 to 3 photons/speckle as a function mean photon density $\bar{k}$. (**a**) Fits from a set of 1000 speckled patterns generated within $\bar{k} = [0.025, 0.25]$ and contrast $C = 0.7$ with Eq. 1. The thin curves represents the contrast boundaries for $C=0$ and $C=1$ respectively. Equation 1 used for fitting *C* is calculated from a weighted average of the fitted $k=1-3$ distribution. The generated and fitted contrasts agrees with each other well. (**b**) Plot of the normalised rms error obtained after repeating 50 times the fitting procedure of (**a**) over newly generated batches of speckle patterns.

Because of the statistical nature of the process, acceptable accuracy can only be obtained by gathering a sufficiently large number of two-pulse speckle patterns. Sets of 1000 patterns are thus simulated for each configuration. Once the patterns are available, the goal is to recover the initial contrast value, using the same analysis tools as for the experimental data. We start by assessing the accuracy in generating sets of 1000 photon maps with the given contrasts (Eq. 1) from the fitting of the photon statistics *P*(*k*) for *k*=1,2, and 3. An example of a fit for a simulated contrast $C(q,\tau )$=0.7 is given in Fig. 2a. The respective uncertainties for *k*=1,2, and 3 are used for the final contrast using a weighted average, though it has recently been shown this can also be accomplished by using the ratio, $R_k = P(k+1)/P(k)$, to solve for a closed-form analytic solution^12^.

To obtain the normalised RMS error reported Fig. 2b, the sets of 1000 photon maps were generated and fitted 50 times for every contrast value in the range [0.1, 1] for 0.1 increments. It was shown that only the lowest contrast value of 0.1 had a deviation larger than 10%.

**Figure 3:**
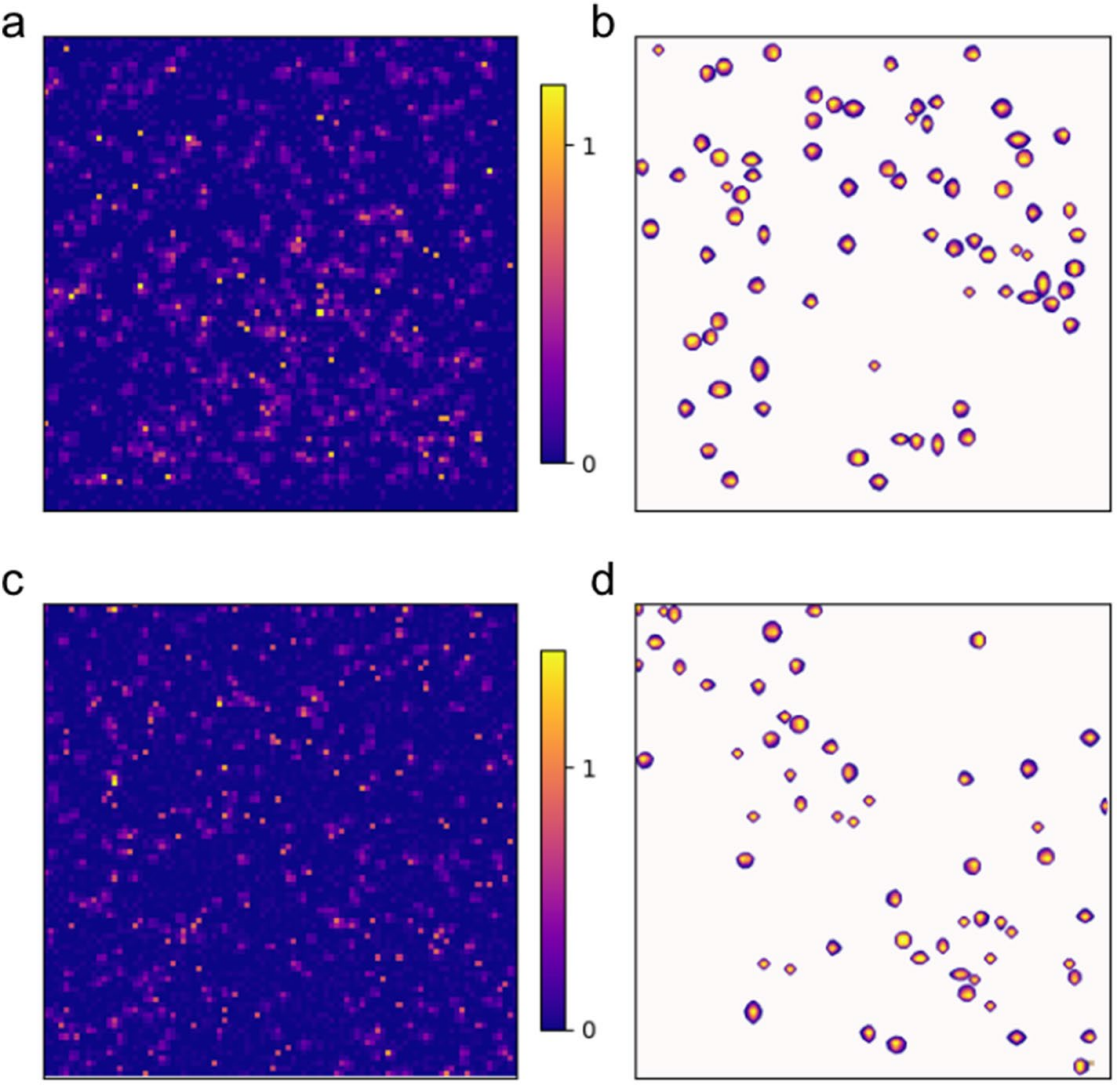
Single charge cloud size analysis from experimental and simulated data. (**a**) Experimental data. (**b**) The single photon events are isolated from (**a**) by selecting connected islands containing a photon readout ($0.5<$photon$<1.5$) and which have a shape fitted with a 2D Gaussian shaped cloud model optimization, Eq. 2. (**c**) Simulated speckle pattern with variable charge cloud sizes. The Gaussian fit shows a variability in single photon sizes (distribution reported in Fig. 4). (**d**) Single photon events isolated and fitted from (**c**).

A cardinal issue with the extraction of the photon statistics is the charge cloud size. In previously published numerical models, the charge cloud size variation was estimated to be null^11^ or as small as 15$\%$ of its average size^13^. Based on the premise that this variation has a major impact on our results, and that droplet algorithms rely on an intimate knowledge of the individual photons, we focus on the investigation of single photon events, comparing experimental and simulated data (see Fig. 3).

For the simplest scenario, droplets with a total intensity that matches the single photon ADU are isolated and fitted with a 2D Gaussian shaped cloud model via a least squares optimization of Eq. 2. In the real experiment however, the variation in the location of the detected photon with respect to the pixel can cause the droplet structure to become complicated rapidly. In the following, the cylindrical symmetry is relaxed and independent widths are considered along the vertical ($\sigma _{x}$) and horizontal ($\sigma _{y}$) directions, and the generated radius is elliptical, i.e. $\sigma _{G}= \sqrt{ \sigma _{x}+\sigma _{y} }$.

**Figure 4:**
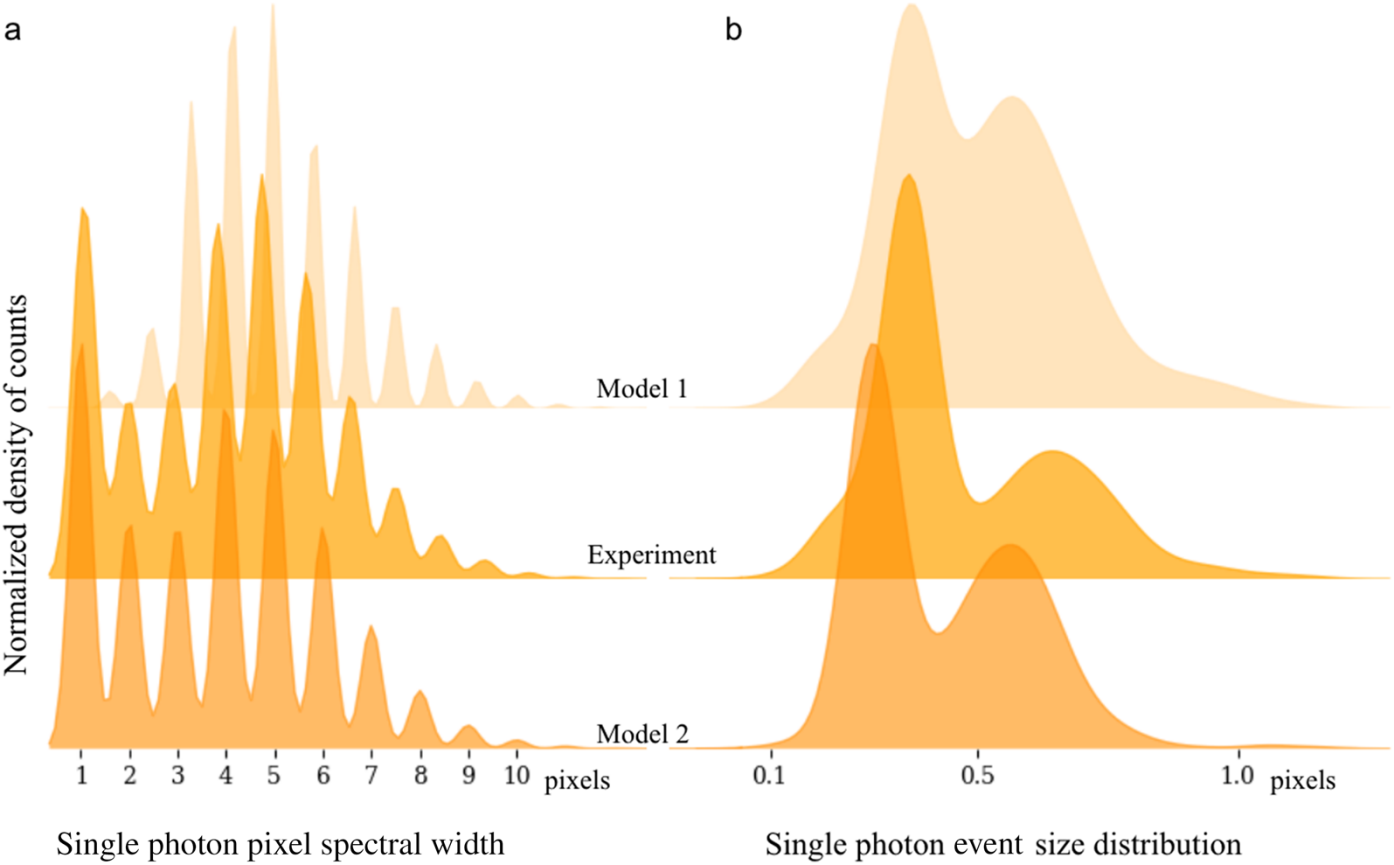
Single charge cloud pixel and size distribution of experimental and simulated data. The analysis described Fig. 3 is repeated over 50 speckle patterns with an applied threshold of 0.05 photon, comparable to experimental readout noise level. (**a**) Two models of charge clouds size distribution (fixed and variable charge cloud size) are compared to experimental data. (**b**) Single photon event size distribution from the Gaussian model in Eq. 2. The simulation based on a weighted photon size distribution reproduces the experimental findings.

The success rate and accuracy of the radius $\sigma _{G}$ fitting optimization was tested first by generating noiseless charge cloud maps with a fixed charge cloud radius ($\sigma _{G}=0.45)$. The convergence success rate obtained was $\sim 90 \%$, with a slightly overestimated average radius $\sigma _{G} \sim 0.5$ along with a narrow distribution that corresponds to the “digitization” of the under-sampled cloud. The accuracy in retrieving the sub-pixel center location ($x_c$, $y_c$) is not the focus of this work. After injection of both a readout noise ($\sigma _N = 0.05$ photon), and a variation of 20$\%$ in the cloud radius (similar to the model but composed from the independent $\sigma _{x,y}$ widths), the fitting convergence rate drops to $\sim 80 \%$. While the recovered average radius remains the same with $\sigma _{G} \sim 0.5$, its distribution becomes very broad, as can be seen in model 1 of Fig. 4b. The “zeroing threshold” was set approximately to the level of noise $\sigma _{G} = 0.05$ photons. However, it is to be noted that the fitted distribution shape and width is dictated by the level of thresholding.

The experimental distribution of $\sigma _{G}$ is reported in Fig. 4b. This knowledge alone does not allow one to further advance the numerical models. Therefore, an additional and essential piece of information is derived from this fit, what we call the single photon pixel spectral width, i.e. the probability for single charge cloud radius pixel spread. This new information is reported in Fig. 4a. A single photon mainly occupies 4 to 6 pixels as expected, with a non-negligible amount of single photons being bounded to a single pixel ($\sim$20 $\%$). This contradicts the original assumptions for model 1, hence this knowledge is reincorporated into a more advanced model 2. To do so, a charge cloud radius is assigned to each of the main probability density peaks from the experimental data in Fig. 4a, along with its corresponding weighted probability $w_i$, as reported in Table 1 below.

For example, a small radius of $\sigma _{G}=0.1$ is appointed to the first peak (1 pixel footprint), with a weighted probability of 20 $\%$, while a large radius $\sigma _{G} = 0.7$ with a low weighted probability of 2.5 $\%$ is attributed to the last of the significant peaks (8 pixels footprint). As can be observed, the output of the new simulation (i.e., model 2), qualitatively recreates the experimental data. Another way to reach a more robust output is an optimization loop. However, the stochastic nature of the charge splitting among CCD pixels^14^ could be an obstacle in that scenario.

**Table 1:** Single photon charge cloud distribution.

Footprint (pixel)	1	2	3	4	5	6	7	8
$\sigma _{G}$	0.1	0.25	0.35	0.45	0.55	0.6	0.65	0.7
$w_i$	0.2	0.125	0.125	0.175	0.175	0.1	0.05	0.025

The table lists the single photon charge cloud distribution in terms of the pixel footprint, corresponding cloud width, and weighted probability for each case, used for model 2.

**Figure 5:**
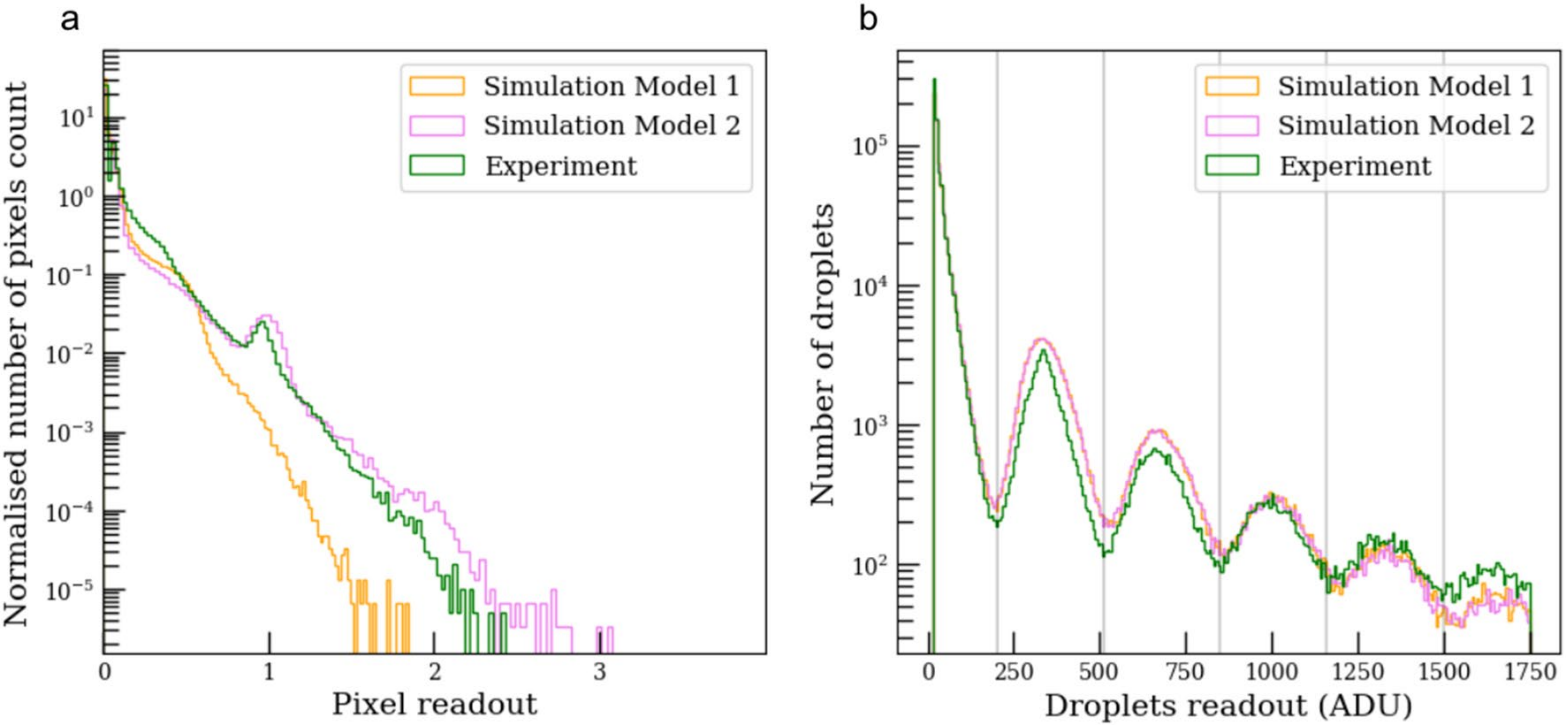
Pixel and droplet histogram for a 1000 experimental and simulated images with the same photon average $\bar{k} = [0.025,0.25]$ and noise readout. (**a**) CCD pixel histogram. Orange and pink are simulations with a fixed and variable photon cloud size. (**b**) Droplet histogram in ADU with peaks corresponding to multiples of a single photon. The vertical lines define the boundaries of the integer number of photons within the droplets, with a maximum of 5 photons per droplet observed.

An additional way to discriminate between the models, is provided from the direct comparison of pixel readout histograms with the experimental data (Fig. 5a). Model 2 clearly reproduces the amplitude of the first photon peak at a nominal readout r  = 340 ADU. The low amplitude of the second photon peak is due to the dilution of the speckles to $3\times 3$ pixels together with the sub-pixel placements, making events containing 2 full photons within a single pixel statistically insignificant. Since readout noise and gain non-uniformity can only modestly influence the fits, it is apparent that the original model 1 is not suitable for this experimental setup.

By repeating the photon droplet exploration without constraining the signal to a single photon, the full extent of the droplet total readout is identified, as seen in the histogram of Fig. 5b. While this last point does not differentiate the models, it gives valuable information to run droplet algorithms. The multiple peaks of the nominal photon readout ($\sim$340 ADU, or 1.19 keV), corresponds to the energy calibration of the detector while the width corresponds to the energy resolution. The photon boundaries denoted by the gray vertical lines determine the integer number of photons within each droplet. The maximum of 5 photons/droplet mainly results from selecting speckle patterns within a limited range of average photon counts per speckle $\overline{k} = [0.025, 0.25]$. Extending the range of $\overline{k}$ results in very large droplets extending over multiple speckles, leading to droplet percolation. Possessing a fully tunable model that reproduces the experimental data (i.e., model 2), we now demonstrate the absolute contrast retrieving abilities of this advanced droplet algorithm.

## Contrast evaluation: results

In this section, the results on contrast extraction using the most straightforward of the droplet algorithms are presented. These aim at converting the raw detector intensity map into a photon count map, accounting for the detector signal-to-noise and charge sharing effects. These algorithms have been thoroughly discussed in the recent work of Sun et al.^13^, where the authors observed that the droplet algorithms commonly used only differ by a constant bias, and hence can be corrected with a linear model. Having a real-time online analysis in mind, we naturally chose the fastest algorithm, the so called Greedy Guess (GG). The Least Squares Fit (LSF)^9^ which rectifies the photon assigned by the GG algorithm is much slower and is usually adapted for an offline analysis scenario.

The GG algorithm processes every speckle pattern in the following manner. In step one, the effects of readout noise are suppressed by “zeroing” the pixels containing values below a given threshold. This operation leaves islands of connected pixels with non-zero signal, identified as droplets by the algorithm. In our study, the connected direction are not discriminated, i.e., diagonal pixels are included to accommodate for the large photons clouds. In the second step, the signal within each identified droplet is summed and ascribed an integer number of photons, according to the readout histograms found in Fig. 5b. Then, the highest pixel value of the droplet is assigned a photon, and a Gaussian cloud centered on that pixel is subtracted from the droplet. The intensity and width of the Gaussian are assigned the mean single photon ADU and the estimated charge cloud size respectively, from which location a Gaussian charge cloud is subtracted whose total intensity is equivalent to the readout of one single photon (Eq. 2). This operation is repeated until the total integer photon count of the droplet reaches zero. Since our GG algorithm operates on the basis of a Gaussian charge cloud model, we name it the Gaussian Greedy Guess (GGG) algorithm. The average photon count/speckle $\bar{k}$ and the photon probabilities *P*(*k*) for $k=1,2,$ and 3 are then computed from the photon maps and fitted with the negative binomial distribution to extract the contrast $C(q,\tau )$ of the speckle pattern.

The single photon ADU, the pixel intensity threshold, and the charge cloud size are thus critical hyper-parameters of the analysis process that can dramatically alter the final result. While the former can often be unambiguously defined from the droplet intensity histogram (Fig. 5), there is not a clear criterion on how to set the other two. This becomes particularly problematic for low signal-to-noise cases, where small variations can have significant effects. With our model, this phase parameter space can now be explored with prior knowledge of the true contrast value, allowing us to define precise rules on how to fix these hyper-parameters for realistic experimental conditions.

**Figure 6:**
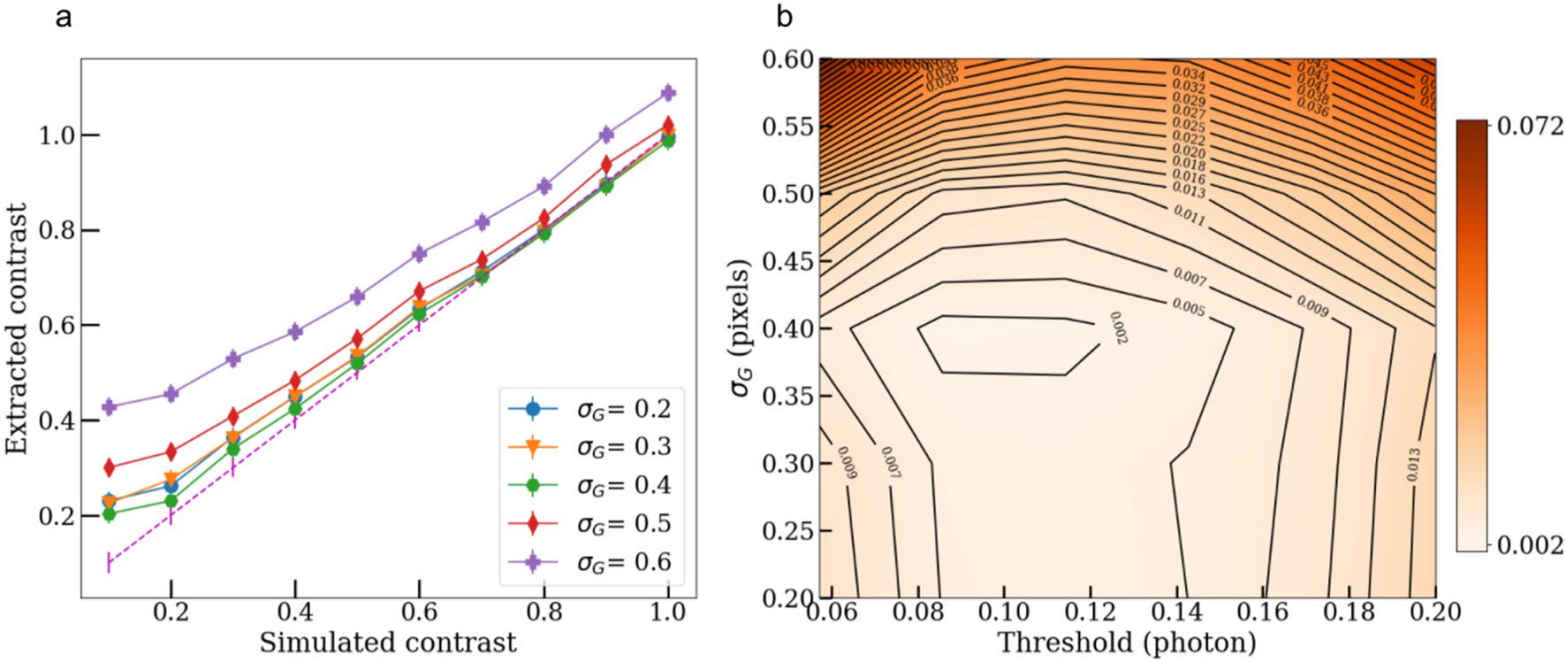
Extracted contrasts from the Gaussian Greedy Guess (GGG) droplet algorithm for a 1000 simulated shots. (**a**) Plot at a selected threshold of 0.1 photon with different size of charge cloud used in GGG. The violet dashed line indicates the ideal/simulated contrasts with error bars based on the r.m.s value calculated in Fig. 2b. (**b**) Surface error metric plot describing what are the optimal hyper-parameters that minimize the distance to the ideal contrasts (r.m.s). The optimal $\sigma _G =0.4$ is smaller than the average ($\sigma _G =0.46$) calculated in Fig. 4b.

Examples of a contrast fit for a pixel intensity threshold of $\sim 0.1$ photon and charge cloud size ranging from $\sigma _{G} = 0.2$ to 0.6 pixels is shown in Fig. 6a. A linear response of the greedy guess is observed, with a slope that tends to 1 as the cloud radius approaches $\sigma _{G} = 0.4$. These two values locate the lowest region of the surface error displayed in Fig. 6b. For every pair of hyper-parameters, the error metric measures the r.m.s distance between the extracted contrasts to the ideal contrasts (dashed magenta line). For error metric values $< 0.025$ (r.m.s), the extracted contrast is deemed absolute (even though the lowest contrast point $C =0.1$ does not fall within the error bars of the ideal reconstructions).

It is not surprising to find that the optimal radius is actually close to the center of mass of the distribution reported in Fig. 4b. Moreover, it is even closer considering the fitting overestimation described for obtaining Fig. 4b. As the thresholding grows too large (> 0.125 photon), the tails of the charge cloud severely peel off and and push the signal distribution towards its center, thus creating a tendency to overestimate the occurrences of multiple photons falling into the same pixel^5^. In addition, to assert that the nominal $16\times 16$ sub-pixel grids provided sufficient resolution of the photon placement, simulations with a larger $32\times 32$ sampling were performed. No change over the accuracy of the contrast extraction was noted. Lowering the resolution further however presumably will affect the results.

Importantly, the degree of average photon count rate per speckle $\bar{k}$ up to which the contrast can be accurately extracted was also investigated. For the sets of simulated data, it is found to be as high as $\bar{k}=0.25$ photon/speckle, a value that approaches the low photon count limit where the speckle statistics formalism has been shown to operate. This is expedient, as accepting a large bandwidth of scattering intensities coming from the untamed SASE pulses, is tantamount to shortening the time to collect a data set at every delay point. Pushing the $\bar{k}$ envelope further is a task worthy of the LSF optimization and its subtle assignment of the photon positions, however it is not guaranteed, as it suffers a separate set of drawbacks^5^.

**Figure 7:**
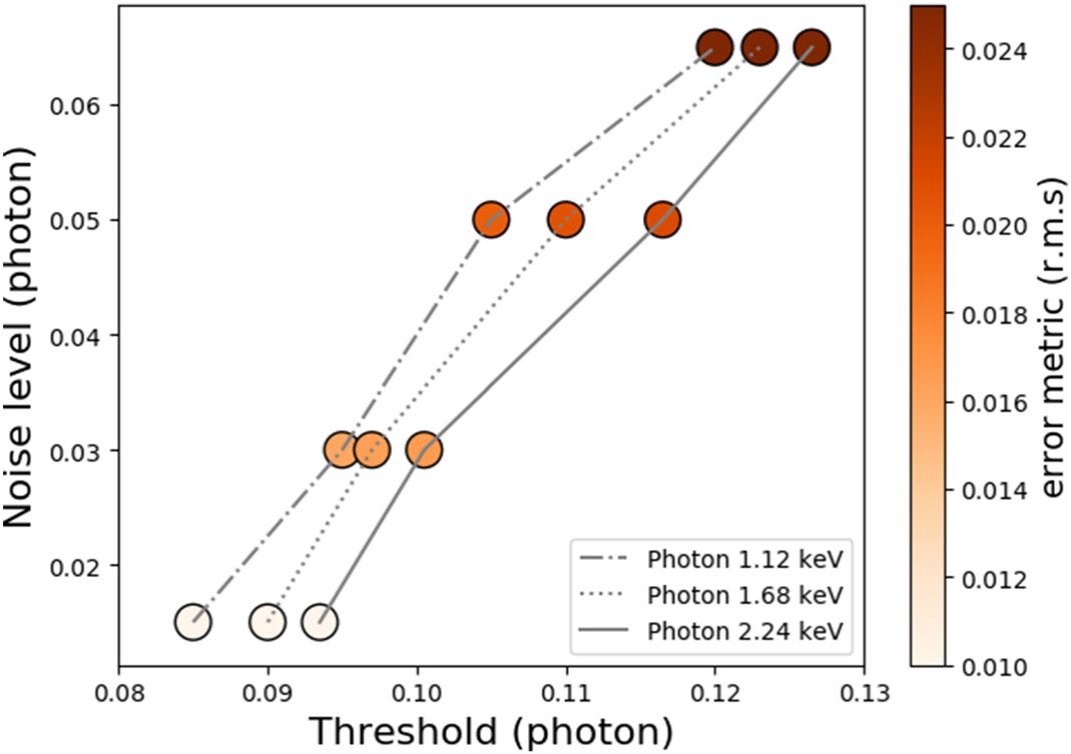
Absolute contrast extraction for different energies (1.12, 1.68, 2.24 keV) and level of noise readout. Only the optimal threshold is reported for the different conditions since its counterpart $\sigma _{G}$ is common to all the results with a value of 0.4. The minimum value of the error metric is color coded for each of the absolute contrast results.

To further generalize the reconstruction parameters, the simulations were extrapolated to higher photon energies with 1.68 and 2.24 keV (525, 700 ADU), respectively. Even though experimental data are not available at these energies yet, the improved SNR can in principle only facilitate this exercise using the GGG droplet algorithm. Conversely, analysis at a lowered photon energy is expected to be far more critical. The CCD pixel readout noise was also varied for every energy with $\sigma _{N}=[ 0.015, 0.03, 0.05, 0.065]$ photons, along with the gain non-uniformity increased up to $\sigma _r$=0.025, as shown in Fig. 7. To obtain the latter figure, we repeated the contrast evaluation procedure used to create Fig. 6b for these 12 different conditions (3 energies, 4 noise levels), and reported the threshold corresponding to each minimum of the surface error metric. Each of these 12 data points demonstrate absolute contrast extraction (< 0.025 r.m.s) over the contrast range 0 to 1 and it can be observed that the optimal thresholding is at the same fraction of the photon energies and also linear with respect to noise ($\sim 0.08 + \sigma _{N}/2$). For its part, $\sigma _{G}$ was found to be common to all the results with a constant value of $\sim 0.4$. Although the charge cloud radius distribution is hypothesized to be about the same for all the simulated photon energies, this tool developed here is dynamic in nature and can be adjusted to address the absolute contrast in other soft x-ray modalities.

## Conclusion

In conclusion, numerical tools were developed to emulate the very distinctive response of a CCD detector to soft x-ray single photon speckle patterns for ultrafast fluctuations studies, which can produce strong charge sharing. The ensuing obstacles were accounted for with a modified fast droplet algorithm, with its set of parameters enabling a linear and non-biased contrast extraction determined for a few soft x-ray photon energies, simulated within the low photon count limit. For a given readout noise, the optimized parameters are energy invariant, with a threshold starting at $\sim 10 \%$ of the photon energy and that slightly increase with the width of the readout noise. The optimal charge cloud radius was found to be located at the adjusted center of mass of its distribution. However, the photon energies were extrapolated up to only twice the experimental photon energy of $\sim$1.12 keV due to a lack of data to compare to at a higher energy range, at which point the charge cloud model may start to substantially deviate. Using an advanced variational Gaussian model, we demonstrate the creation of simulations which match the experimental data and perform a droplet analysis which enables absolute contrast extraction. Notably, the intrinsic pulse energy fluctuations due to the SASE process at the XFEL were considered and shown not to affect the photon determination accuracy up to a large fraction of the low photon limit (where the sample dynamics are not influenced). Furthermore, this motivates the possibility for a real-time analysis of XPFS data at XFELs in the future.
